# Survivin as a Novel Biomarker in the Pathogenesis of Acne Vulgaris and Its Correlation to Insulin-Like Growth Factor-I

**DOI:** 10.1155/2016/7040312

**Published:** 2016-10-10

**Authors:** Hanan A. Assaf, Wafaa M. Abdel-Maged, Bakheet E. M. Elsadek, Mohammed H. Hassan, Mohamed A. Adly, Soher A. Ali

**Affiliations:** ^1^Department of Dermatology, Faculty of Medicine, Sohag University, P.O. Box 82524, Sohag, Egypt; ^2^Department of Biochemistry and Molecular Biology, Faculty of Pharmacy, Al-Azhar University, Assiut Branch, P.O. Box 71524, Assiut, Egypt; ^3^Department of Biochemistry and Molecular Biology, Qena Faculty of Medicine, South Valley University, P.O. Box 83523, Qena, Egypt; ^4^Department of Zoology, Faculty of Science, Sohag University, P.O. Box 82524, Sohag, Egypt

## Abstract

Survivin, a member of the inhibitor of apoptosis protein family, has an important role in cell cycle regulation. Insulin-like growth factor-I (IGF-I) is a polypeptide hormone with wide range of biologic effects including stimulation of lipogenesis in sebaceous glands. Their overexpression in some fibrotic disorders suggests a possible implication of both IGF-I and survivin in the pathogenesis of acne and/or acne scars. The current study aimed to assess and correlate serum levels of IGF-I and survivin in patients with active acne vulgaris and postinflammatory acne scars and to evaluate their lesional expressions in comparison to healthy controls. Serum IGF-I and survivin were estimated using commercially available ELISA kits and their tissues expressions were investigated using Western blotting. Our findings suggest that IGF-I and survivin could play potential roles in the pathogenesis of active acne vulgaris and more importantly in postinflammatory acne scars with significant positive correlation coefficient between serum levels of IGF-I and survivin which support IGF-I-/PI3K-/AKT-mediated downregulation of nuclear expression of FoxO transcription factors resulting in enhanced survivin expression.

## 1. Introduction

Acne vulgaris is a chronic inflammatory disease of the pilosebaceous unit, characterized by seborrhea, formation of comedones, erythematous papules, and pustules and less frequently by nodules, deep pustules, or pseudocysts [1]. The primary and the pathognomonic lesion of acne is microcomedo, a microscopic lesion invisible to the eye, which evolves commonly into inflammatory or noninflammatory lesions. The formation of microcomedo requires complex interplay of altered follicular keratinization, hyperplasia of sebaceous glands, and overcolonization of sebaceous glands with Propionibacterium acnes [2].

Among adolescents, acne has prevalence over 90% [3] and persists into adulthood in approximately 12%–14% of cases with psychological and social implications [4]. In some cases, acne is accompanied by scarring, a consequence of abnormal resolution or wound healing following the damage that occurs in the sebaceous follicle during acne inflammation [5]. The scarring process can occur at any stage of acne [6].

During the last decades, there is increasing evidence in support of the interplay of insulin-like growth factor-I (IGF-I) signaling during puberty, which may have a causal role in pathogenesis of acne by influencing adrenal and gonadal androgen metabolism that was reported to be an inducer of sebum production through sterol response element-binding proteins [7]. IGF-I, also called somatomedin C, is a small polypeptide hormone with an approximate molecular weight of 7 kDa [8]. It mediates its effects through the IGF-I receptor (IGF-IR) that belongs to the tyrosine kinase family of growth factor receptors [9]. IGF-IR autophosphorylation subsequent to IGF-I binding initiates downstream signaling pathways that mediate a wide variety of intracellular signaling pathways to regulate glucose transport, protein synthesis, cell proliferation, and survival in many cells and tissues [10].

In the skin, IGF-I is synthesized mainly by dermal fibroblasts and melanocytes and also possibly by keratinocytes of the stratum granulosum [11]. Additionally, it was reported that IGF-I has distinct effects on sebocyte growth, differentiation, and proliferation [12, 13]. It has been localized to the peripheral cells of sebaceous glands in the rat [14]. In human skin appendages, the strongest expression of IGF-I protein was found in maturing sebocytes and suprabasal cells of sebaceous ducts. This pattern of expression suggests a role for IGF-I as a sebaceous mitogen and morphogen [15]. However, studies of the role of IGF-I signaling in skin development and function have been largely limited by the fact that IGF-IR-null mice die soon after birth, and there is therefore no model available for studies on the direct effects of IGF-IR on skin development in function* in vivo* [16].

Recent studies have shown that elevated levels of serum IGF-I correlate with overproduction of sebum and acne [17] as a result of IGF-I and insulin induced lipogenesis of sebaceous glands, probably by induction of sterol response element-binding protein-1 (SREBP-1) [12]. Also, it was reported that, in the skin, IGF-I is produced by dermal fibroblasts to stimulate its proliferation and increases mRNA levels of procollagen I [18]; hence it was implicated in the pathogenesis and progression of many fibrotic disorders [19]. Furthermore, its elevated levels in patients with systemic sclerosis and morphoea confirm the possible role of IGF-I in development of fibrosis in acne scar [20, 21].

Survivin, a member of inhibitors of the apoptosis (IAP) gene family, is a 16.5 kDa protein that inhibits apoptosis and regulates cell division, proliferation, and survival [22]. The expression of survivin is undetectable or is found at very low levels in normal tissues, whereas it is found at relatively higher levels in various malignant tissues, embryonic and fetal tissues, and also few normal adult tissues, including skin [23]. In human skin, survivin function has long remained unclear because of several works showing the absence of survivin in human adult epidermis. However, more recent reports have shown that survivin is indeed expressed in normal human skin, and it is localized in the cytoplasm of a few cells located in the basal layer of the epidermis [24].

Up to date, the biological functions of survivin, other than its antiapoptotic effect, are not well understood in human [25]. However, many studies have defined its role in regulating functions of several normal adult cells suggesting that survivin disruption could have adverse consequences [26]. Interestingly, survivin was not previously evaluated, studied, described, or investigated in the fibrosis progression of the cutaneous tissues but its upregulation has already been described in certain liver diseases and during hepatic stellate cells activation. This proves to large extent the potential role of survivin in fibrogenesis process, most likely through regulation of apoptosis [27, 28]. Therefore, additional studies on the expression and subcellular localization of survivin in relation to function will confirm its key role in the skin and will open the field to new therapeutic strategies for many cutaneous conditions such as acne scaring.

## 2. Materials and Methods

### 2.1. The Study Population

The current study had been conducted between June 2014 and May 2015 after the approval by Research Committee at Faculty of Medicine, Sohag University (R. Nr. 14/15/06/2014). The study included 45 subjects that were randomly selected from those attending the Dermatology Outpatient's Clinic at Sohag University Hospital, Faculty of Medicine, Sohag University, Sohag, Egypt. Prior to initiation of the study, every subject was informed about the aim of the study and was given a written consent for participation. They were classified into three groups; the first group included 15 patients suffering from active acne and did not receive any treatment for 3 months; the second group included 15 patients suffering from postinflammatory acne scars with no history of previous skin resurfacing, no active infection, and with no use of oral isotretinoin in the previous 6 months; and the third group consisted of 15 healthy subjects to serve as a control group. The patients and controls are matched in age and body mass index (BMI). Patients with a history of human immunodeficiency virus (HIV), chronic or acute hepatitis, liver cirrhosis, and benign or malignant tumors, as well as any other kind of cutaneous or fibrotic lesions were excluded. From each subject, 5 mL blood was collected into a separator gel tube for serum isolation which was subsequently divided into aliquots and stored in a frozen matter at −20°C for later analysis.

Also, skin punch biopsies (4 mm) were taken from the back of each subject after being locally anesthetized with 2% lidocaine at the Dermatology Department of Sohag University Hospital and homogenized in ice-cold Tris-HCl lysis buffer, pH 7.4, containing 1% protease inhibitor cocktail (Cell Signaling Technology, Inc., MA, USA) using Potter-Elvehjem rotor-stator homogenizer (glass/teflon homogenizer), fitted with a teflon pestle and stored in a frozen matter at −70°C for subsequent assessment of tissue IGF-I and survivin expression by Western blotting technique.

### 2.2. ELISA Assays of IGF-I and Survivin

Quantitative determinations of serum IGF-I and survivin were achieved using corresponding commercially available ELISA kits from Assaypro LLC., (MO, USA) and Biospes Co., Ltd., (Chongqing, China), respectively, according to the manufacturer's instructions.

### 2.3. Western Blotting Assessments of IGF-I and Survivin Expression

In the skin tissue homogenates, proteins in each corresponding sample were denatured at 95°C for 5 minutes in 2x Laemmli buffer followed by addition of 5% 2-mercaptoethanol. SDS-PAGE electrophoresis was achieved by loading 50 *μ*g protein per lane at 75 volts through resolving gel (18% for IGF-I and 14% for survivin) followed by 125 volts during approximately 2 hours and transferred to a PVDF membrane using T-77 ECL semidry transfer unit (Amersham BioSciences UK Ltd) for 2 hours. Immunoblotting was performed by incubating the PVDF membrane in TBS buffer containing 0.1% Tween and 5% nonfat milk for one hour at 4°C, followed by overnight incubation at 4°C with rabbit anti-survivin polyclonal antibody (Bioss Inc., MA, USA) and mouse anti-IGF-I monoclonal antibody (Novus Biologicals, LLC, Littleton, CO, USA) at a dilution of 1 : 1500. After being washed three times with TBST buffer, each membrane was incubated for 1 hour at room temperature with an alkaline phosphatase-conjugated goat anti-mouse secondary antibody (Novus Biologicals, LLC, Littleton, CO, USA) at a dilution of 1 : 5000. After being washed four times in TBST, the membrane bound antibody was detected with a commercially available BCIP/NBT substrate detection Kit (Genemed Biotechnologies, Inc., CA, USA). Equivalent protein loading for each lane was confirmed by stripping and reblotting each membrane at 4°C against mouse monoclonal anti-*β*-actin antibody (Santa Cruz Biotechnology, Inc., CA, USA) at a dilution of 1 : 5000. The analysis was repeated to assure reproducibility of results.

### 2.4. Statistical Analysis

Statistical analyses of the data were carried out using GraphPad Prism version 5.0 (Graphpad Software, Inc., CA, USA). Data comparisons were performed using analysis of variance (ANOVA) followed by Tukey's *t*-test. The levels of significance were accepted with *p* < 0.05 and all relevant results were graphically displayed as mean ± SD. The correlations between the continuous variables were performed using Pearson's correlation coefficient. Categorical variables were expressed as percentages.

## 3. Results

### 3.1. The Study Population

The current study included 30 patients (15 males and 15 females) with an average age of 24.76 ± 8 years (19–29.7 years). Those patients were divided into two groups; the first group included 15 patients (8 males and 7 females) with an average age of 24.0 ± 7 years that were suffering from untreated active acne lesions on the face (in 100% of patients), back (in 100% of patients), chest (in 92.8% of patients), upper limbs (in 13.3% of patients), and abdomen (in 6.6% of patients). The second group included 15 patients (7 males and 8 females) with an average age of 24.6 ± 9.2 years that were suffering from infections free postinflammatory acne scars affecting the face (in 100% of patients), back (in 6.6% of patients), chest (in 6.6% of patients), upper limbs (in 6.6% of patients), and abdomen (in 6.6% of patients). The study included another third group consisting of 15 healthy age and sex matched subjects (8 males and 7 females) to serve as a control group with an average age of 27.4 ± 13 years (18–32 years).

### 3.2. Biochemical and Molecular Assessments of IGF-I and Survivin

**Figure 1:**
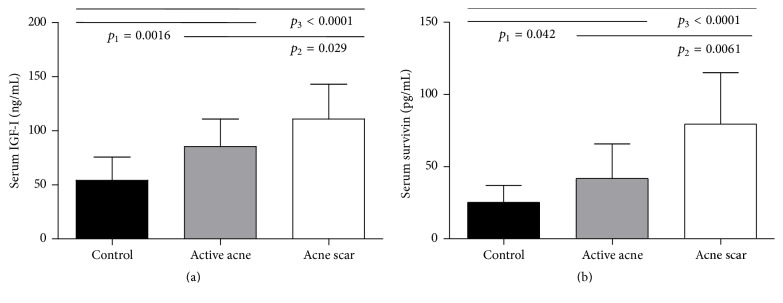
Serum levels of IGF-I (a) and survivin (b) in the study groups as achieved by ELISA. Data are presented as mean ± SD (*n* = 15). *p*_1_ refers to the statistical difference between the control group and the active acne group; *p*_2_ refers to the statistical difference between the active acne group and the acne scar group; *p*_3_ refers to the statistical difference between the control group and the acne scar group. One-way ANOVA was used for testing significance. *p* value of < 0.05 was considered significant.

The results of the current study showed that serum levels of IGF-I were significantly elevated in active acne (*p* < 0.01) and acne scar (*p* < 0.001) groups in comparison to the healthy control group. Also, IGF-I serum levels showed significant increase in acne scar group compared to active acne group (*p* < 0.05) (Table 1 and Figure 1(a)). Western blotting assessments showed marked overexpression of IGF-I in the skin tissues from acne scar group than its corresponding levels in the active acne group and the control group with mild overexpression of IGF-I in the skin tissues from active acne group than its corresponding levels in the control group (Figure 3(a)).

**Figure 2:**
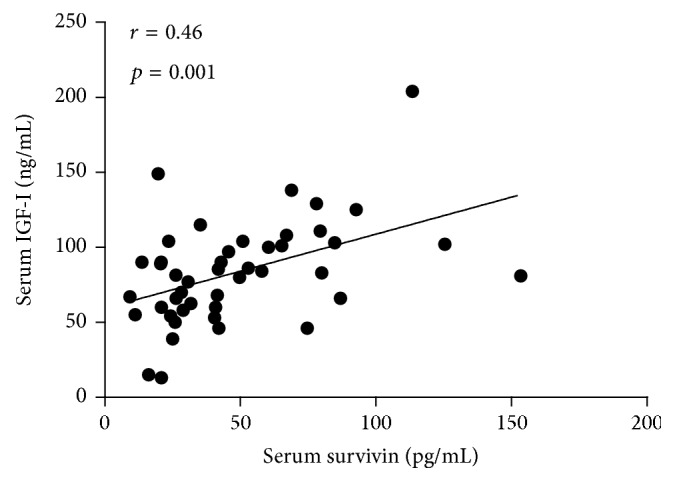
Pearson correlation between serum levels of IGF-I (ng/mL) and survivin (pg/mL), showing positive correlation.

**Figure 3:**
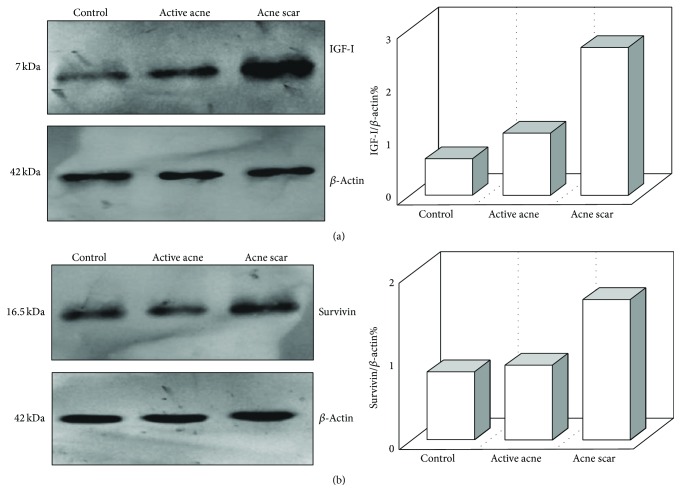
Expression of IGF-I (a) and survivin (b) in the skin tissue homogenates as achieved by Western blotting. *β*-Actin was used in parallel as internal control. The right panels represent corresponding quantification of each gel analysis measured by Image J software and expressed as a *β*-actin ratio, showing higher lesional expressions of IGF-1 and survivin in the acne scar group than in the active acne group and the control group, and also lesional expressions of IGF-I in the active acne group were higher than in the control group.

Furthermore, this study showed that serum survivin levels were significantly increased in active acne (*p* < 0.05) and acne scar (*p* < 0.001) groups in comparison to the healthy control group. These serum survivin levels were significantly higher in acne scar group than in the active acne group (*p* < 0.01) (Table 1 and Figure 1(b)). Western blotting assessments showed that survivin expression was stronger in the acne scar group than in the active acne group and the control group (Figure 3(b)). Additionally, the current study revealed a significant positive correlation coefficient between serum levels of IGF-I and survivin (*r* = 0.46 and *p* = 0.001) (Figure 2).

## 4. Discussion

The etiology of acne vulgaris appears to be multifactorial, involving follicular hyperkeratinization, proliferation of Propionibacterium acnes, increased sebum production, and inflammation [29]. Our results have shown that the circulating levels of IGF-I were obviously elevated in the active acne and acne scar groups in comparison to the healthy control group with a remarkable increase in acne scar group compared to active acne group (Table 1 and Figure 1(a)). Western blotting assessments in this study showed marked overexpression of IGF-I in the lesional skin tissues from acne scar group than its corresponding levels in the active acne group and the control group with mild overexpression of IGF-I in the lesional skin tissues from active acne group than its corresponding levels in the control group (Figure 3(a)).

The source of increased IGF-I in acne skin may be derived either from the systemic circulation or from local IGF-I biosynthesis of acne-prone skin areas. These results were in agreement with the reports of several other investigators who estimated the circulating and local tissue levels of IGF-I in acne patients using ELISA and immunohistochemistry technique, respectively [7, 17, 30–32]. Also, there is indirect evidence in favor of the predominant effect of systemic IGF-I in the pathogenesis of acne. Acne is primarily a disease of puberty associated with increased somatotropic signaling. Pituitary gland-derived growth hormone (GH) stimulates IGF-I synthesis in the liver, the major tissue source of IGF-I synthesis in the human body. Untreated patients with Laron syndrome having congenital systemic IGF-I deficiency due to a GH-receptor defect never develop acne [33], unless substituted with high doses of recombinant IGF-I [34].

A testimony of this hypothesis has been mentioned since years when it was reported that IGF-I acts as a key hormone mediator for the synthesis of androgens from the adrenals and gonads, or even within the skin itself, amplifying cutaneous androgen activity and enhancing proliferation of sebaceous follicles [7]. It can play this role through enhancing the gene expression of steroidogenic enzymes that are responsible for converting cholesterol into steroid precursors in human sebaceous glands for local androgen production [30]. Stimulation of steroidogenic enzymes by IGF-I is most likely mediated through sterol response element-binding proteins-1 (SREBPs-1), a nucleotide sequences found in the promoter regions of several lipogenic genes in the cholesterol and fatty acid biosynthesis pathways. IGF-I has been found to increase lipogenesis in an experimental sebocyte model, accompanied by an increase in expression of SREBPs-I mRNA and protein [35]. IGF-I was also observed to increase lipid production in sebocytes* in vitro* via the activation of IGF-I receptors through multiple pathways including activation of the phosphoinositide-3-kinase (PI3K) pathway causing an increase in sebaceous lipogenesis, sebocytes, and keratinocytes proliferation, which can eventually aggravate acne. Meanwhile, activation of PI3K pathway was reported to reduce the nuclear content of the transcription factor FoxO1 deficiency, the key nutrigenomic regulator of acne target genes. Nuclear FoxO1 deficiency has been linked to all major factors of acne pathogenesis, that is, androgen receptor transactivation, comedogenesis, increased sebaceous lipogenesis, and follicular inflammation [36, 37]. Thus, the systemic route of IGF-I delivery to the sebaceous follicle plays a crucial role for IGF-I-dependent FoxO regulation. Sebaceous glands are surrounded by a capillary vasculature such as the terminal hair follicle, which appears to deliver systemic IGF-I and other hormones such as circulating androgens. Systemic overproduction of IGF-I increases adrenal and gonadal androgen synthesis and operates thus in another fashion. Both IGF-I and androgens stimulate sebaceous lipogenesis [36]. However, in another study, there was no statistically significant difference in the circulating levels of IGF-1 between the acne patients and the controls [30].

The obviously noticed increase in the serum and tissues levels of IGF-I in acne scar group compared to active acne group and control group could suggest that IGF-I has greater role in the pathogenesis of acne scar fibrous tissue than in active acne which is multifactorial. This is in agreement with previous studies in which IGF-I was found to be increased or overexpressed in other diseases associated with fibrotic disorders such as morphoea [20], idiopathic pulmonary fibrosis [38], and oral submucous fibrosis [19]. It seems that increased IGF-I level in acne scar is secondary to increased local lesional production, and the severity of sclerotic process is related most probably to the level of local IGF-I production as within the lesion of morphoea [20]. A possible explanation for these observations is that IGF-I can stimulate the proliferation of fibroblasts and increases mRNA levels of procollagen type I [39]. Another contradicting study reported that serum IGF-I levels were within the normal range in patients with some fibrotic disorders such as progressive systemic sclerosis [40]. The authors suggested that the role of IGF-I in the pathogenesis of progressive systemic sclerosis might be at the fibroblast receptor level or in response of fibroblasts to IGF-I [40].

The present study aimed also to estimate the circulating levels and the expression pattern of survivin, the smallest member of IAP family that serves a critical function in cell cycle progression especially in actively dividing cells, in the active acne and the acne scar groups in comparison to the healthy control group. Our results revealed that the serum levels of survivin were markedly increased in the active acne and the acne scar groups in comparison to the healthy control group with an obvious increase in acne scar group compared to active acne group (Table 1 and Figure 1(b)). Western blotting assessments in this study showed that survivin expression was stronger in the acne scar group than in the active acne group and the control group (Figure 3(b)). This is in agreement with a previous study which found an increased survivin in keratinocytic proliferative and inflammatory states, which are deeply involved in the pathogenesis of the acne lesions [29, 41]. Notably, increased survivin may affect both the sebaceous gland (sebocyte survival) and the perifollicular dermal tissue (scar formation). Nuclear survivin expression has been observed in sebaceous hyperplasia and neoplasia [42]. To the best of our knowledge, serum and tissue levels of survivin were not evaluated in patients with acne scar in previous studies. So, we compare the role of survivin in fibrosis with other fibrotic diseases. For instance, increased survivin expression was found to contribute in fibroblast apoptosis resistance in idiopathic pulmonary fibrosis [28]. In another study that employed a murine model of reversible liver fibrosis, survivin expression was found to be significantly increased during the establishment of fibrosis. Moreover, the resolution phase of this fibrosis model, which is dependent on hepatic satellite cell (myofibroblast) apoptosis, coincided with the reduction of survivin expression to prefibrosis level [43]. This demonstrates that survivin expression could have a mechanistic link with the pathogenesis of fibrosis in fibrotic disorders including acne scarring process and thereby suppression of its expression could be a choice for the reversal of these lesions.

This study showed also a significant positive correlation coefficient between serum levels of IGF-I and survivin (*r* = 0.46  and  *p* = 0.001) (Figure 2). Increased IGF-I signaling is related to the enhanced survivin expression by IGF-I-/PI3K-/AKT-mediated downregulation of nuclear expression of FoxO proteins such as FoxO1 and FoxO3a. The survivin promoter is regulated by FoxO1 and FoxO3a [44, 45]. Thus, there is compelling translational evidence that increased insulin/IGF-I signaling in acne with deficient nuclear availability of FoxO transcription factors may enhance survivin expression. Accordingly, a study done by Melnik proposed that acne vulgaris is related to overactivation of mTORC1 signaling [46]. In fact, Monfrecola research group provided experimental evidence for increased mTOR expression and S6K1 phosphorylation (and thus increased mTORC1 signaling) in acne group compared to acne-free healthy controls [47], which was also recently proved by other researchers [48].

Remarkably, it is known from androgen-dependent prostate epithelial cells that IGF-I-mediated activation of mTORC1 upregulates survivin expression in prostate epithelial cells [49]. It is noteworthy to mention that the sebocyte and the prostate epithelial cell are both responsive to androgen and IGF-I growth signals. Taken together, decreased nuclear FoxO signaling and increased mTORC1 signaling are most likely the explanation for increased survivin in acne lesional skin.

The present findings are an important piece in the exciting puzzle of acne pathogenesis, which is primarily driven by increased production of “acne sebum” that exposes the sebofollicular duct to proinflammatory lipids, such as free palmitic acid, oleic acid, and sapienic acid. The role of proinflammatory lipid in the initiation of acne lesions has recently been emphasized [50].

It is well known that the most effective antiacne drug that most potently suppresses sebum production is oral isotretinoin, which functions via TRAIL-mediated induction of apoptosis [51]. In contrast, survivin is an antiapoptotic protein. Its expression depends on stimulation of the kinases Akt and mTORC1, which attenuates cell apoptosis. Melnik recently emphasized that proinflammatory sebocyte growth and survival signaling in acne vulgaris is reversed by proapoptotic isotretinoin signaling [52]. Thus, prosurvival/survivin signaling in acne is attenuated via isotretinoin-mediated sebocyte apoptosis.

## 5. Conclusions

In conclusion, these data collectively demonstrate that both IGF-I and survivin play an important role in the pathogenesis of active acne vulgaris and more importantly in the pathogenesis of postinflammatory acne scar fibrotic tissue formation. This may shed new light on nontraditional strategies for the future medical treatments of acne vulgaris as well as preventing postinflammatory acne scaring, through regulation of IGF-I and survivin expression.

## 6. Recommendations

Additional more comprehensive large-scale studies on the expression and subcellular localization of IGF-I and survivin are recommended to determine their correlation with the severity of these dermatological ailments. Future studies should clarify whether sebaceous gland IGF-I and survivin expressions are upregulated in sebaceous cell hyperplasia of acne vulgaris and whether isotretinoin treatment downregulates mTORC1, IGF-I, and survivin expression.

## Figures and Tables

**Table 1:** Serum levels of IGF-I and survivin in the studied groups.

	Control	Active acne	Acne scar
Serum IGF-I (ng/mL)	54.1 ± 21.5	85.4 ± 25.3^*∗∗*^	110.8 ± 32.3^*∗∗∗*,†^
Serum survivin (pg/mL)	25.2 ± 11.8	41.8 ± 23.9^*∗*^	79.4 ± 35.6^*∗∗∗*,††^

Data are presented as mean ± SD (*n* = 15). *∗* and † indicate significant changes from control and active acne groups respectively. *∗* and † indicate significant change at *p* < 0.05; *∗∗* and †† indicate significant change at *p* < 0.01; *∗∗∗* indicates significant change at *p* < 0.001. There were significant higher serum levels of IGF-I and survivin in acne scar and active acne groups than in the control group.
